# *In vitro* activity of pertuzumab in combination with trastuzumab in uterine serous papillary adenocarcinoma

**DOI:** 10.1038/sj.bjc.6605448

**Published:** 2009-11-17

**Authors:** K El-Sahwi, S Bellone, E Cocco, M Cargnelutti, F Casagrande, M Bellone, M Abu-Khalaf, N Buza, F A Tavassoli, P Hui, D-A Silasi, M Azodi, P E Schwartz, T J Rutherford, S Pecorelli, A D Santin

**Affiliations:** 1 Department of Obstetrics, Gynecology and Reproductive Sciences, Yale University School of Medicine, New Haven, CT, USA; 2 Department of Internal Medicine and Oncology, Yale University School of Medicine, New Haven, CT, USA; 3 Department of Pathology, Yale University School of Medicine, New Haven, CT, USA; 4 Department of Obstetrics and Gynecology, Division of Gynecologic Oncology, University of Brescia, Brescia, Italy

**Keywords:** uterine serous papillary cancer, pertuzumab, trastuzumab, endometrial carcinoma

## Abstract

**Background:**

Uterine serous papillary adenocarcinoma (USPC) is a rare but highly aggressive variant of endometrial cancer. Pertuzumab is a new humanised monoclonal antibody (mAb) targeting the epidermal growth factor type II receptor (HER2/neu). We evaluated pertuzumab activity separately or in combination with trastuzumab against primary USPC cell lines expressing different levels of HER2/neu.

**Methods:**

Six USPC cell lines were assessed by immunohistochemistry (IHC), flow cytometry, and real-time PCR for HER2/neu expression. *c-erbB2* gene amplification was evaluated using fluorescent *in situ* hybridisation (FISH). Sensitivity to pertuzumab and trastuzumab-induced antibody-dependent cell-mediated cytotoxicity (ADCC) and complement-dependent cytotoxicity (CDC) was evaluated in 5 h chromium release assays. Pertuzumab cytostatic activity was evaluated using proliferation-based assays.

**Results:**

Three USPC cell lines stained heavily for HER2/neu by IHC and showed amplification of the *c-erbB2* gene by FISH. The remaining FISH-negative USPCs expressed HER2/neu at 0/1+ levels. In cytotoxicity experiments against USPC with a high HER2/neu expression, pertuzumab and trastuzumab were similarly effective in inducing strong ADCC. The addition of complement-containing plasma and interleukin-2 increased the cytotoxic effect induced by both mAbs. In low HER2/neu USPC expressors, trastuzumab was more potent than pertuzumab in inducing ADCC. Importantly, in this setting, the combination of pertuzumab with trastuzumab significantly increased the ADCC effect induced by trastuzumab alone (*P*=0.02). Finally, pertuzumab induced a significant inhibition in the proliferation of all USPC cell lines tested, regardless of their HER-2/neu expression.

**Conclusion:**

Pertuzumab and trastuzumab induce equally strong ADCC and CDC in FISH-positive USPC cell lines. Pertuzumab significantly increases tratuzumab-induced ADCC against USPC with a low HER2/neu expression and may represent a new therapeutic agent in patients harbouring advanced/recurrent and/or refractory USPC.

Endometrial cancer is the most common female genital tract malignancy in the United States, with an incidence of 40 100 new cases and 7470 deaths annually (Jemal *et al*, 2008). Two subtypes of endometrial carcinoma, namely, type I and type II tumours, have been described, on the basis of both clinical and histopathological variables. Type I endometrial cancers, which account for the majority of cases, are usually well differentiated and endometrioid in histology. These neoplasms are frequently diagnosed in younger women, are associated with a history of hyper-oestrogenism as the main risk factor, and typically have a favourable prognosis with appropriate therapy. In contrast, type II endometrial cancers are poorly differentiated tumours, often with serous papillary (USPC) or clear cell histology (Sorosky, 2008).

Uterine serous papillary adenocarcinomas are highly aggressive tumours with propensity for lymphovascular invasion and intraperitoneal and extra-abdominal spread, even at presentation (Creasman *et al*, 2001). Although they account for only a minority of endometrial cancers (i.e., <10%), about 40% of all relapses occur in this group of patients. Furthermore, USPC tumours that overexpress the HER2/neu receptor have a worse prognosis than those that do not. Our group, as well as others, including large cooperative studies reported by the Gynecologic Oncology Group, has previously shown that up to 50% of USPC patients may overexpress HER2/neu in their tumour, and these tumours may be more biologically aggressive than cancers that do not overexpress the receptor (Santin *et al*, 2002; Slomovitz *et al*, 2004; Santin *et al*, 2005a, 2005b; Diaz-Montes *et al*, 2006; Villella *et al*, 2006; Grushko *et al*, 2008).

The *erbB2* gene encodes for HER2/neu, a member of the erbB receptor tyrosine kinase family. This is a family of four transmembrane glycoproteins (erbB1, erbB2, erbB3, and erbB4) that are expressed on epithelial, mesenchymal, and neuronal cells (Yarden and Sliwkowski, 2001). Ligand binding results in dimerisation of the receptor either with a twin receptor (homodimerisation) or with one of its siblings (heterodimerisation) (Yarden and Sliwkowski, 2001). This leads to phosphorylation of intracellular tyrosine kinase residues that serve as docking sites for various effectors and transcription factors that ultimately modulate various biological responses, such as proliferation, survival, migration, and differentiation. It is noteworthy that the HER2/neu heterodimer is characterised by a stronger and more diverse signalling potential than other erbB dimers (Yarden and Sliwkowski, 2001). Importantly, HER2/neu overexpression has been previously reported to be associated with cancer cell proliferation, poor survival, and resistance to therapy in multiple human tumours (Slamon *et al*, 1987; Berchuck *et al*, 1990, 1991; Hetzel *et al*, 1992; Santin *et al*, 2005).

Pertuzumab (Omnitarg, Genentech, South San Francisco, CA, USA), the first of a new class of agents designated as HER dimerisation inhibitors, is a humanised IgG1 monoclonal antibody (mAb) that sterically binds domain II of the erbB2 receptor. Unlike trastuzumab (Herceptin), the principal mechanism of action of which is believed to be through recruiting host immune cells (natural killer (NK) cells) and setting off an antibody-dependent cell-mediated cytotoxicity (ADCC) process (Clynes *et al*, 2000; Gennari *et al*, 2004; Arnould *et al*, 2006), pertuzumab is believed to inhibit a wider array of downstream signal transduction pathways through inhibition of lateral signal transduction, that is, heterodimerisation. Several *in vitro* and *in vivo* studies conducted on a variety of human tumour cell lines have clearly elucidated this mechanism of action (Mullen *et al*, 2007; Agus *et al*, 2002; Jackson *et al*, 2004; Nahta *et al*, 2004; Takai *et al*, 2005). To our knowledge, however, a therapeutic potential for pertuzumab has, thus far, not been reported in endometrial cancer management.

In this study, to evaluate the potential clinical activity of pertuzumab when used alone or in combination with trastuzumab, we tested six USPC primary cell lines for HER2/neu receptor expression and *c-erbB2* gene amplification, and evaluated the sensitivity of these biologically aggressive tumours expressing different levels of HER2/neu to pertuzumab- and trastuzumab-mediated ADCC and complement-dependent cytotoxicity (CDC) in standard 5 h chromium release assays. The potential *in vitro* growth inhibition of pertuzumab, trastuzumab, and a combination of the two mAbs in USPC cell lines was also studied.

## Methods

### Establishment of USPC cell lines

Primary USPC tumour cell lines from six patients with invasive USPC were obtained from fresh tumour biopsy samples collected at the time of surgery, under approval of the Institutional Review Board. Tumour samples were collected from patients who experienced rapid tumour progression during adjuvant chemotherapy after primary surgical debulking. Tumours were staged according to the International Federation of Gynecologists and Obstetricians operative staging system. Patient characteristics are described in Table 1. Six primary USPC cell lines (USPC ARK-1, USPC ARK-2, USPC ARK-3, USPC ARK-4, USPC ARK-5, and USPC ARK-6) were established after sterile processing of tumour samples from surgical biopsy specimens, as described previously for ovarian carcinoma specimens (Santin *et al*, 1996). Primary USPC cell lines with limited passages (i.e., <50) were analysed by flow cytometry for HER2/neu expression immediately after tumour processing and after *in vitro* culture from 1 week to 3 years.

### Immunostaining of formalin-fixed tumour tissues and cell blocks obtained from primary USPC

Formalin-fixed, paraffin-embedded tissue blocks from the six USPC patients from whom primary cell lines were established were retrieved from surgical pathology files. Specimens were reviewed by a surgical pathologist. The level of expression of HER2/neu was then evaluated on the most representative block by standard immunohistochemical staining, using the Hercept test (Dako, Glostrup, Denmark). Briefly, immunohistochemical stainings were performed on 4-*μ*m sections of formalin-fixed, paraffin-embedded tissue. After pretreatment with 10 mM citrate buffer at pH 6.0 using a steamer, they were incubated with anti-HER-2/neu polyclonal Ab (Dako) at a 1 : 200 dilution. Slides were subsequently labelled with streptavidin-biotin (LSAB; Dako), stained with diaminobenzidine, and counterstained with haematoxylin.

Similarly, cell cultures from all six primary cell lines used in cytotoxicity experiments were trypsinised, and cells were suspended in a cytorich fixative (Richard Allen Scientific, Kalamazoo, MI, USA) and centrifuged for 5 min at 2650 r.p.m. The supernatant was removed without disturbing the cell button. Four drops of human plasma and four drops of thromboplastin (Simplastin Excel, Biomerieux, Durham, NC, USA) were added to resuspend the cell button. Specimens were set aside until a clot formed (generally 5 min). The clot was then placed in a mesh bag, fixed in 10% buffered formalin, and processed as per the routine histological technique. HER2/neu immunohistochemical stainings were performed on paraffin-embedded 5 *μ*m sections of cell blocks after deparaffinisation and rehydration, using the HercepTest kit (Dako) according to the manufacturer's instructions. HER2/neu intensity of staining was graded as 0 (negative=no staining, or membrane staining in <10% of tumour cells), 1+ (light staining=a faint/barely perceptible partial membrane staining in >10% of tumour cells), 2+ (moderate staining=a weak-to-moderate membrane staining in >10% of tumour cells), or 3+ (heavy staining=a strong complete membrane staining in >10% of tumour cells). Appropriate positive and negative controls were used with each case.

### FISH of cell blocks obtained from primary USPC

Fluorescent *in situ* hybridisation (FISH) analysis was performed using the PathVysion Her-2 DNA FISH Kit (Abbott Molecular Inc., Abbott Park, IL, USA) according to the manufacturer's instructions. Briefly, 5 *μ*m sections of cell blocks were deparaffinised and rehydrated, followed by acid pretreatment and proteinase K digestion. A probe mix containing an orange probe directed against the *Her2* gene (Vysis, Inc., Downers Grove, IL, USA, LSI Her2/neu) and a green probe directed against the pericentromeric region of chromosome 17 (Vysis CEP 17) were added and specimens were denatured for 5 min at 73 °C. The slides were then incubated overnight in a humidity chamber at 37 °C. On the following day, the slides were washed and a fluorescence mounting medium containing 4,6-diamidino-2-phenylindole (DAPI) was applied. Fluorescent signals in at least 30 non-overlapping interphase nuclei with intact morphology were scored using a Zeiss Axioplan 2 microscope (Carl Zeiss Meditec, Inc., Dublin, CA, USA) with a × 100 planar objective, using a triple band-pass filter that permits simultaneous blue, green, and red colours. Tumour cells were scored for the number of red (HER2/neu) and green (chromosome 17) signals. A case was scored as amplified when the ratio of the number of fluorescent signals of HER2/neu to chromosome 17 (R/G) was ⩾2. The ploidy status of chromosome 17 was determined by calculating the average number of copies of chromosome 17 on the basis of the count of 30 cell nuclei. Disomy was defined as a chromosome 17 number of 1.5–2.5. Such a range took into account that even a given tumour specimen with predominantly disomy 17 could still show some deviation from an average of two signals per cell, because of the genetic instability of the tumour, high mitotic index, and potential nuclear truncation through tissue sectioning. Chromosome 17 polysomy was defined as a chromosome 17 average number of >2.5. Monosomy was defined as a chromosome 17 average number of <1.5.

### Flow cytometry

The clinically marketed anti-HER/neu mAb pertuzumab (Omnitarg; Genentech Inc) was used for our study. For comparison, we also used the anti HER/neu trastuzumab (Herceptin; Genentech). Both pertuzumab and trastuzumab are humanised mAbs of the G1 type that bind, with high affinity, the extracellular domain of the HER/neu receptor (Mr 185 000 transmembrane glycoprotein). Whereas pertuzumab binds domain II, trastuzumab binds domain IV. For staining, a fluorescein isothiocyanate-conjugated goat antihuman F(ab1)2 immunoglobulin was used as a secondary reagent (BioSource International, Camarillo, CA, USA). Analysis was conducted with a FACScalibur, using cell Quest software (BD Biosciences, San Diego, CA, USA).

### Quantitative real-time–PCR

RNA isolation from all six primary USPC cell lines used in cytotoxicity experiments was performed using TRIzol Reagent (Invitrogen, Carlsbad, CA, USA), according to the manufacturer's instructions. Quantitative PCR was carried out with a 7500 Real-Time PCR System using the manufacturer's recommended protocol (Applied Biosystems, Foster City, CA, USA) to evaluate the expression of erbB2 in all samples. Each reaction was run in duplicate. Briefly, 5 *μ*g of total RNA from each sample was reverse transcribed using SuperScript III first-strand cDNA synthesis (Invitrogen). Five microlitres of reverse-transcribed RNA samples (from 500 *μ*l of total volume) were amplified using the TaqMan Universal PCR Master Mix (Applied Biosystems) to produce PCR products specific for erbB2. The primers and probe for erbB2 were obtained from Applied Biosystems (Assay ID Hs00170433_m1). The comparative threshold cycle (*C*_T_) method (Applied Biosystems) was used to determine gene expression in each sample, relative to the value observed in the lowest non-malignant endometrial epithelial cell sample, using glyceraldehyde-3-phosphate dehydrogenase (Assay ID Hs99999905_m1) RNA as internal control.

### Tests for ADCC

A standard 5 h chromium (^51^Cr) release assay was performed to measure the cytotoxic reactivity of Ficoll-Hypaque-separated peripheral blood lymphocytes (PBLs) from several healthy donors in combination with trastuzumab and/or pertuzumab against USPC target cell lines. The release of ^51^Cr from target cells was measured as described (Santin *et al*, 1999), as evidence of tumour cell lysis after exposure of tumour cells to various concentrations of trastuzumab and pertuzumab (ranging from 1 to 100 *μ*g ml^−1^) and different target/effector cell ratios. Controls included the incubation of target cells alone or with PBLs or mAb separately. The chimeric anti-CD20 mAb rituximab (Rituxan; Genentech) was used as control in all bioassays. Antibody-dependent cell-mediated cytotoxicity was calculated as the percentage of killing of target cells observed with mAb plus effector cells as compared with ^51^Cr release from target cells incubated alone.

### Test for complement-mediated target cell lysis and *γ*-globulin inhibition

A standard 5 h chromium (^51^Cr) release assay identical to that used for ADCC assays, except that human plasma (as a source of complement) diluted 1 : 2 was added in place of effector cells, was used to test for complement-mediated target cell lysis. To test for the possible inhibition of ADCC against USPC cell lines by physiological human plasma concentrations of *γ*-globulin, heat-inactivated (56 °C for 30 min) human plasma was diluted 1 : 2 before being added in the presence or absence of effector PBLs. In some experiments, non-heat-inactivated human plasma (diluted 1 : 2) was added in the presence of effector PBLs. Controls included the incubation of target cells alone or with either lymphocytes or mAb separately. Rituximab was used as control mAb.

### IL-2 enhancement of ADCC

To investigate the effect of interleukin-2 (IL-2) on pertuzumab-mediated ADCC, effector PBLs were incubated for 5 h at 37°C at a final concentration of IL-2 (Aldesleukin; Chiron Therapeutics, Emeryville, CA, USA) ranging from 50 to 100 IU ml^−1^ in 96-well microtitre plates. Target cells were primary USPC cell lines exposed to pertuzumab (concentrations ranging from 1 to 100 *μ*g ml^−1^), whereas controls included the incubation of target cells alone or with PBLs in the presence or absence of IL-2 or mAb, respectively. Rituximab was used as control mAb. ADCC was calculated as the percentage of killing of target cells observed with mAb plus effector PBLs, as compared with target cells incubated alone.

### Proliferation assay

To evaluate the potential cytostatic effect of pertuzumab on primary USPC cell lines, cells were seeded in a 96-well plate at a density of 2000–5000 cells per well in RPMI 1640 medium (Invitrogen) with 10% foetal bovine serum (Gemini, Woodland, CA, USA). After 24 h, pertuzumab, trastuzumab, and a 1 : 1 combination of both antibodies were added at a final concentration of 20 *μ*g ml^−1^. The final volume of medium per well was set at 100 *μ*l. After 48–72 h, proliferation was evaluated using CellTiter 96 Aqueous One Solution cell proliferation assay (Promega, Madison, WI, USA), as directed by the manufacturer. Results of this colorimetric assay were interpreted as percentage inhibition of proliferation compared with control cells growing in culture medium devoid of antibodies or in the presence of rituximab.

### Statistical analysis

For quantitative real-time PCR (qRT–PCR) data, right skewing was removed by considering copy-number ratios relative to the lowest-expressing NEC sample (‘relative copy numbers’), log_2_-transforming them to ΔCTs, and comparing the results using an unequal-variance *t*-test for the USPC-*vs*-NEC difference. Group means with 95% confidence intervals (CIs) were calculated by computing them on ΔCTs and reverse transforming the results to obtain means (95% CIs) of relative copy numbers. Differences in the HER2/neu expression by flow cytometry were analysed by an unpaired *t*-test, and a *P*-value <0.05 among samples was considered to be significant. The Wilcoxon rank–sum test was used to compare USPC types to normal endometrium for differences in immunohistochemistry (IHC) staining intensities. Sample-type differences were expressed as odds ratios accompanied by 95% CIs. The Kruskal–Wallis test and *χ*^2^ analysis were used to evaluate the differences in HER2/neu-induced ADCC levels and inhibition of proliferation in primary tumour cell lines. Statistical analysis was performed using SPSS version 15 (SPSS, Chicago, IL). A *P*-value of <0.05 was considered as being statistically significant.

## Results

### HER2/neu expression by IHC in USPC tissue blocks and primary USPC cell lines

**Figure 1:**
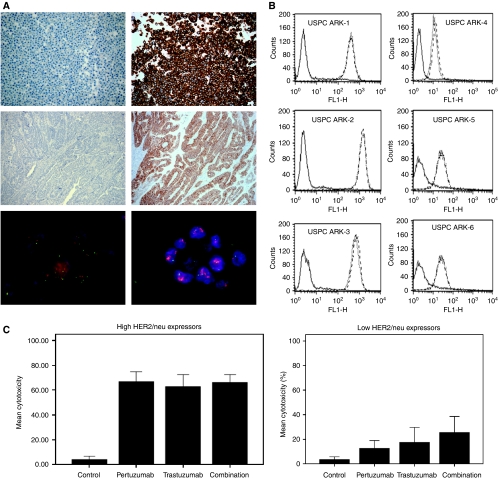
(**A**) Upper panel: Representative HER2/neu expression by immunohistochemistry (IHC) in uterine serous papillary adenocarcinoma (USPC) cell blocks. Left: USPC ARK-4 shows negative staining for HER2/neu; Right: USPC ARK-3 shows strong (3+) staining for HER2/neu. Middle panel: Representative HER2/neu expression by IHC in USPC tissue blocks. Left: USPC ARK-6 shows low/negative staining for HER2/neu; Right: USPC ARK-2 shows strong (3+) staining for HER2/neu. Lower panel: Representative example of negative (USPC ARK-6, left) *vs* positive (USPC-ARK2, right) fluorescent *in situ* hybridisation (FISH) amplification of the *c-erbb2* gene. Tumour cells were scored for the number of red (HER2/neu) and green (chromosome 17) signals as described in the Methods section. (**B**) Flow cytometry histograms of primary USPC cell lines showing high (USPC ARK-1, USPC ARK-2, and USPC ARK-3) and low (USPC ARK-4, USPC ARK-5, and USPC ARK-6) expression of HER2/neu. Rituximab-anti-CD20 control antibody (solid line); trastuzumab (dashed line); pertuzumab (dotted line). (**C**) Left panel: Antibody-dependent cytotoxicity (mean±s.d.) in USPC cell lines with high HER2/neu expression (i.e., USPC ARK-1, -2, and -3). Average percentage cytotoxicity in high expressors from six independent matched experiments, showing significantly higher cytotoxicity with trastuzumab (the Kruskall–Wallis test and *χ*^2^ analysis, *P=*0.001), pertuzumab (*P=*0.0001), and combination of pertuzumab and trastuzumab (*P=*0.001) when compared with either peripheral blood lymphocytes (PBLs) only or control antibody rituximab. E : T, effector to target cell ratio. Right panel: Antibody-dependent cytotoxicity (mean±s.d.) in USPC cell lines with a low HER2/neu expression (i.e., USPC ARK-4, -5, and -6). Average percentage cytotoxicity in low expressors from 12 independent experiments, showing significantly higher cytotoxicity with trastuzumab (*P*<0.01), pertuzumab (*P=*0.01), and combination of pertuzumab and trastuzumab (*P=*0.01) when compared with either PBLs only or control antibody rituximab. Combination of pertuzumab and trastuzumab (total dose 2.5 *μ*g ml^−1^) induced significantly higher cytotoxicity (*P=*0.001) when compared with either trastuzumab (2.5 *μ*g ml^−1^) or pertuzumab (2.5 *μ*g ml^−1^) alone. E : T, effector to target cell ratio.

Using formalin-fixed paraffin blocks of USPC tissues from which the six primary tumour cell lines were established, HER2/neu expression was detected by IHC (i.e., score from 1+ to 3+) in all six samples. Three samples showed a moderate-to-strong membrane staining (i.e., 2+, 3+), whereas the remaining three showed weak staining (1+) (Figure 1A and Table 2). To determine whether the high, moderate, or low expression of the HER2/neu receptor detected on USPC tissue blocks may differ in the primary USPC cell lines because of the potential selection of a sub-population of cancer cells present in the original tumour, or whether *in vitro* expansion conditions may have modified receptor expression, we performed additional immunohistochemical analysis of HER2/neu on the cell blocks obtained from all six primary USPC culture cell lines used in cytotoxicity experiments. Strong membrane staining for HER2/neu (3+) in cell blocks was confirmed by IHC in USPC ARK-1 and USPC ARK-2 cell lines. USPC ARK-3 was found to express HER2/neu at the 3+ level on the cell block of cultured tumour cells, whereas on the original tissue block, HER2/neu was reported at the 2+ level (Table 2, Figure 1A). USPC ARK-6 showed weak membrane staining (1+) in the cell block and in the original tissue block. One score difference in staining intensity was noted between paraffin-embedded tumour tissues and cell blocks obtained from the remaining two cell lines (i.e., USPC ARK-4 and USPC ARK-5) (Table 2). This minor discrepancy in scoring USPC with low HER2/neu expression is likely to be related to test reproducibility and/or inter-observer variability.

### HER2/neu expression by qRT–PCR in USPC

Uterine serous papillary adenocarcinoma cell lines grown as primary cultures were tested by qRT–PCR to confirm at mRNA level the different HER2/neu surface receptor expressions. Normal endometrial samples were used as negative controls. mRNA copy number for USPC ARK-1, USPC ARK-2, and USPC ARK-3 (i.e., high HER2/neu expressors by IHC) were 373, 607, and 677, respectively. This was compared with an mRNA copy number of 7, 13, and 6 in USPC ARK-4, USPC ARK-5, and USPC ARK-6 (i.e., low HER2/neu expressors by IHC), respectively (Table 2). These data are in full agreement with the results obtained by IHC and flow cytometry.

### *erbB2* gene amplification by FISH and ploidy in USPC

Fluorescent *in situ* hybridisation was performed on formalin-fixed paraffin-embedded tissue blocks from four USPCs, as well as on the cell blocks obtained from all six primary cell lines used in cytotoxicity experiments. Test results are shown in Table 2. *erbB2* gene amplification was detected in USPC ARK-1, USPC ARK-2, and USPC ARK-3, suggesting that the strong receptor expression and high HER2/neu mRNA level are likely caused by gene amplification. In contrast, USPC ARK-4, USPC ARK-5, and USPC ARK-6 were found to be negative for *erbB2* gene amplification (Table 2). When the chromosome 17 average copy number was calculated, polysomy was identified in four primary cell lines (i.e., USPC ARK-1, USPC ARK-4, USPC ARK-5, and USPC ARK-6), whereas disomy was evident in the two remaining cell lines (i.e., USPC-ARK-2 and USPC-ARK-3).

### HER2/neu expression by flow cytometry in primary USPC

The HER2/neu receptor expression was evaluated by FACS analysis on all six primary USPC cell lines using trastusumab, pertuzumab, and a combination of the two mAbs. In addition, as negative controls, several B-cell lines (EBV-transformed lymphoblastoid B cell lines) established from the same USPC patients from whom the tumour cell lines had been established were also studied. Very high reactivity against HER2/neu was found in 100% of primary USPC ARK-1, USPC ARK-2, and USPC ARK-3 cell lines using either pertuzumab or trastuzumab (mean fluorescence intensity (MFI) ranging from 387 to 1381) (Figure 1B, Table 3). Significantly lower HER2/neu expression was observed in USPC ARK-4, USPC ARK-5, and USPC ARK-6 (i.e., MFI range 13 to 31) (Figure 1B, Table 3). These findings obtained with viable (i.e., not fixed) tumour cells are consistent with the IHC results obtained on the tissue block and fixed cell lines and confirm a higher expression of HER2/neu in our first three cell lines compared with the latter ones. HER2/neu expression in all USPC samples was higher than that in negative controls (data not shown). Importantly, in matched experiments, we found the combination of pertuzumab and trastuzumab to consistently provide a significantly higher MFI for HER2/neu in six out of six (100%) USPC cell lines when compared with the HER2/neu MFI results obtained using either antibody alone (Table 3). These results are likely to be related to the fact that trastuzumab and pertuzumab recognise two different epitopes on the surface of USPC cell lines.

### USPCs are resistant to NK activity but sensitive to pertuzumab-mediated ADCC

All six primary USPC cell lines available were tested for their sensitivity to NK cytotoxicity when challenged with heterologous PBLs collected from several healthy donors in a standard 5 h ^51^Cr release assay (Table 4). USPC cells were consistently found to be resistant to NK-mediated cytotoxicity when combined with PBLs at E : T ratios varying from 25 : 1 to 50 : 1 (range of cytotoxicity from 0.8 to 10% with all E : T ratios). Similarly, USPC cell lines incubated with the rituximab (2.5 *μ*g ml^−1^) control antibody endured no significant cytotoxicity (range from 0.3 to 8.6%).

We then investigated the sensitivity of USPC cell lines to heterologous PBLs in the presence of pertuzumab (2.5 *μ*g ml^−1^), trastuzumab (2.5 *μ*g ml^−1^), and a combination of both (Table 4). In cell lines expressing high levels of erbB2 (i.e., USPC ARK-1, USPC ARK-2, and USPC ARK-3), high cytotoxicity was achieved with pertuzumab (mean±s.d., 61±25.6%; range, 32.5–67.3%; *P=*0.0001) and trastuzumab (mean±s.d., 56.3±14.2%; range, 32.5–77.1%; *P=*0.0001) compared with control, that is, either PBLs alone (mean±s.d., 3.2±5.6%; range, 0–19%) or PBLs and rituximab (mean±s.d., 2.5±3.8%; range, 0–13%), in multiple experiments. No significant difference in cytotoxicity was found between the two antibodies in high HER2/neu expressors (Table 4). The upper panel of Figure 1C depicts the mean cytotoxicity induced by pertuzumab, trastuzumab, and the combination of both mAbs in high-expressor USPC cell lines.

In cell lines expressing low levels of erbB2 (i.e., USPC ARK-4, USPC ARK-5, and USPC ARK-6), significant cytotoxicity was achieved with pertuzumab (mean±s.d., 14±11%; range, 0.4–32%) compared with either PBLs alone (mean±s.d., 4±4%; range, 0–13%; *P=*0.001) or PBLs and rituximab (mean±s.d., 2.4±1.8%; range, 0.2–4.8%; *P=*0.001). Similarly, significant cytotoxicity was also achieved with trastuzumab (mean±s.d., 21±18%; range, 3–56%) compared with either PBLs alone (mean±s.d., 4±4%; range, 0–13%; *P=*0.015) or PBLs and rituximab (mean±s.d., 2.4±1.8%; range, 10.2–4.8%; *P=*0.018). Trastuzumab induced significantly higher cytotoxicity than pertuzumab in matched experiments (*P=*0.01). Both pertuzumab and trastuzumab produced significantly higher cytotoxicity (mean±s.d., 32±18%; range, 9–66%*)* when compared with PBLs (*P=*0.001) or PBLs and rituximab (*P=*0.001) (Table 4). Importantly, when pertuzumab and trastuzumab were used in combination against USPC with a low HER2/neu expression, the cytotoxicity achieved was significantly higher than that achieved by either pertuzumab (*P=*0.001) or trastuzumab (*P=*0.01) used alone (Table 4, Figure 1C, lower panel). This implies that the combination of trastuzumab and pertuzumab may potentiate the induction of ADCC against USPC that expresses low levels of HER2/neu.

### Effect of complement and endogenous IgG on pertuzumab-mediated ADCC against USPC

**Figure 2:**
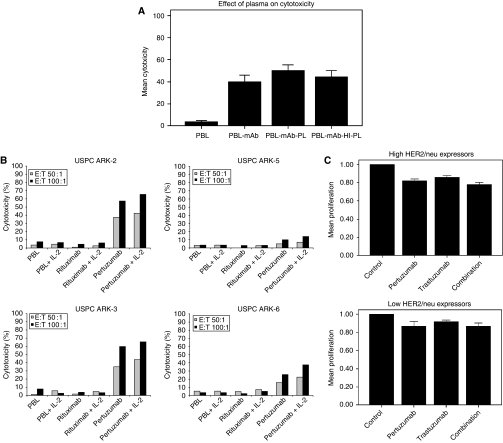
(**A**) Representative effect of human plasma (diluted 1 : 50) on cytotoxicity induced by PBLs in a total of 13 independent experiments in five uterine serous papillary adenocarcinoma (USPC) cell lines including three high HER2/neu expressors and two low expressors. Addition of plasma (diluted 1 : 2) to PBL in the presence of pertuzumab caused a significant increase in cell lysis (mean±s.d., *P=*0.01), attributable to complement-mediated cytotoxicity. Inactivation of complement by heating annihilated this effect. PBL, peripheral blood lymphocytes; mAb, monoclonal antibody; PL, plasma; HI PL, heat-inactivated plasma. (**B**) Representative examples of interleukin-2 (IL-2) enhancement of antibody-dependent cell-mediated cytotoxicity (ADCC) mediated by pertuzumab (2.5 *μ*g ml^−1^) against two high (i.e., USPC-ARK-2 and -3) and two low (i.e., USPC-ARK-5 and -6) HER2/neu expressor USPC cell lines. ^51^Cr-labelled USPC cells (10 000 cells per sample) were incubated in the presence of 100 IU ml^−1^ of IL-2 for 5 h. Effector PBL with medium only or in the presence of rituxan (2.5 *μ*g ml^−1^) was used as control. Pertuzumab-mediated ADCC was significantly enhanced in both high and low HER2/neu expressor USPC cell lines when compared with controls (*P=*0.04). Similar results were obtained after incubation of effector PBL with 50 IU ml^−1^ of IL-2 (data not shown). (**C**) Representative effect of pertuzumab, trastuzumab, and a combination of the two mAbs on the proliferation of USPC cell lines with a high (upper panel) and low (lower panel) Her2/neu expression. Tumour cells were seeded in 96-well plates (2500–3000 cells per well) in the presence of pertuzumab (20 *μ*g ml^−1^), trastuzumab (20 *μ*g ml^−1^), or a combination of both antibodies (total concentration 20 *μ*g ml^−1^, ratio 1 : 1) as described in the Methods section. After 72 h, proliferation was measured using CELLTITER 96 AQueous One Solution Cell Proliferation Assay (Promega). Data are presented as mean±s.d. of a total of 33 independent experiments. Both pertuzumab and trastuzumab induced a significant reduction in the proliferation of cells when compared with control cells (the Kruskal–Wallis test and *χ*^2^ analysis, *P=*0.0001). Pertuzumab alone and the combination of pertuzumab and trastuzumab were significantly more effective than trastuzumab alone (*P=*0.03).

To evaluate primary USPC cell lines for their sensitivity to complement-mediated cytotoxicity and to evaluate possible inhibition of ADCC by endogenous IgG, five USPC cell lines (i.e., three high HER2/neu expressors, namely, USPC ARK-1, USPC ARK-2, and USPC ARK-3; and two low HER2/neu expressors, namely, USPC ARK-5 and USPC ARK-6) were challenged with human plasma diluted 1 : 2 (with and without heat inactivation) in the presence and absence of effector cells and pertuzumab in standard 5 h ^51^Cr release assay. The addition of untreated plasma in the absence of PBL with or without pertuzumab was unable to induce significant cytotoxicity against any of the USPC cell lines (data not shown). These data illustrate the lack of significant cytotoxicity mediated by complement proteins in the absence of effector cells. Furthermore, the addition of endogenous IgG (i.e., heat-inactivated plasma, diluted 1 : 2) to PBLs in the presence of pertuzumab did not significantly alter the degree of ADCC. In contrast, as shown in Figure 2A, the addition of untreated plasma (diluted 1 : 2) to PBLs in the presence of pertuzumab consistently increased ADCC against all USPC primary cell lines regardless of their expression of HER2/neu (i.e., pertuzumab+plasma: mean±s.d., 50±17%; range, 14–71% *vs* pertuzumab alone: mean±s.d., 40±21%; range, 13–71%; *P=*0.01). Similarly, pertuzumab in combination with plasma induced significantly higher cytotoxicity when compared with PBLs treated with plasma (*P=*0.001) or PBLs treated with plasma in the presence of rituximab (*P=*0.001). Taken together, these results show that pertuzumab induces CDC in USPC cell lines.

### IL-2 enhancement of ADCC against USPC

To investigate the effect of low doses of IL-2 in combination with pertuzumab (2.5 *μ*g ml^−1^) on ADCC against USPC cell lines, PBLs from healthy donors were incubated for 5 h in the presence of 50–100 IU ml^−1^ of IL-2. As representatively shown in Figure 2B for all USPC cell lines tested (i.e., two high HER2/neu expressors, namely, USPC ARK-2 and USPC ARK-3; and two low HER2/neu expressors, namely, USPC ARK-5 and USPC ARK-6), significantly higher cytotoxicity was evident when pertuzumab was combined with IL-2 (mean±s.d., 46.9±11.%; range, 22.4–74.2%) *vs* the use of pertuzumab alone (mean±s.d., 41.5±12.8%; range, 15.8–71.9%; *P=*0.04). Similarly, pertuzumab in combination with IL-2 induced significantly higher cytotoxicity when compared with PBLs treated with IL-2 (*P=*0.03) or PBLs treated with IL-2 in the presence of rituximab (*P=*0.03). In contrast, no significant increase in cytotoxicity was detected after 5 h of IL-2 treatment in the absence of pertuzumab or in the presence of rituximab control mAb (Figure 2B). These results suggest that low levels of IL-2 may enhance ADCC mediated by pertuzumab *in vitro* in both high and low HER2/neu expression USPC cell lines.

### Pertuzumab inhibits *in vitro* proliferation of USPC cell lines

Inhibition of proliferation by pertuzumab was tested *in vitro* in all six primary USPC cell lines endowed with a high and low HER2/neu expression. The effect of pertuzumab (20 *μ*g ml^−1^) was compared with that of trastuzumab (20 *μ*g ml^−1^) and with a combination of both antibodies (total concentration 20 *μ*g ml^−1^, ratio 1 : 1). Cell proliferation was significantly inhibited in the presence of pertuzumab, trastuzumab, and the combination of the two mAbs in all USPC cell lines tested, with the percentage of inhibition varying from 4 to 59% (pertuzumab), 3 to 48% (trastuzumab), and 7 to 63% (mAbs combination) in multiple experiments (*P*<0.001) (Figure 2C). Pertuzumab was significantly more effective than trastuzumab in inhibiting the proliferation of USPC cell lines regardless of their high or low HER2/neu expression (*P*<0.001). The combination of pertuzumab and trastuzumab was more cytostatic when compared with that of trastuzumab used alone against all cell lines (Figure 2C, *P*<0.005). Finally, a combination of pertuzumab and trastuzumab was significantly more cytostatic against the high Her2/neu expressor cell lines when compared with pertuzumab used alone (Figure 2C, *P*<0.01).

## Discussion

Our group has recently reported HER2/neu overexpression by IHC and amplification of the *c-erbB2* gene by FISH in a large percentage of patients harbouring USPC (Santin *et al*, 2002; Santin *et al*, 2005a, 2005b). These findings, which have been recently confirmed by other groups (Diaz-Montes *et al*, 2006), as well as those in cooperative multicentric studies reported by the Gynecologic Oncology Group (Grushko *et al*, 2008), have highlighted the potential of targeting HER2/neu as a new therapeutic marker in patients harbouring aggressive type II carcinomas such as USPC. Similar to the great promise for treatment of metastatic breast cancer in patients harbouring tumours with proven amplification or strong (i.e., 3+ by IHC) HER2/neu overexpression, endometrial cancer patients with disease refractory to standard treatment modalities may potentially benefit from HER2/neu-targeted therapy. Consistent with this view, in this study we demonstrated that HER2/neu is highly expressed in three out of the six primary USPC cell lines established in our laboratory. Overexpression of the HER2/neu receptor was demonstrated by IHC and flow cytometry, and was also correlated with both mRNA expression and gene amplification by qRT–PCR and FISH, respectively.

In a previous study, we reported significant trastuzumab-induced ADCC in USPC cell lines showing strong (i.e., 3+) HER2/neu overexpression (Santin *et al*, 2002). In this study, we carefully analysed and compared the potential cytotoxic activity of pertuzumab with that of trastuzumab in multiple USPC cell lines expressing different levels of HER2/neu. In addition, we studied and compared the effect of the combination of pertuzumab and trastuzumab in eliciting ADCC against these tumours. Our results showed for the first time high sensitivity to pertuzumab-induced ADCC in all six primary USPC cell lines available in this study. In this regard, when USPC showing *erbB2* gene amplification by FISH was challenged by PBLs and pertuzumab in a standard 5 h chromium (^51^Cr) release assay, significant levels of cytotoxicity, comparable with those achieved by trastuzumab, were consistently observed.

Although *in vivo* and *in vitro* studies have proposed several mechanisms of action for IgG1 mAbs, such as trastuzumab and pertuzumab, including receptor downmodulation and downstream signal inhibition, ADCC has evolved as an instrumental mechanism to explain the clinical activity of these mAb (Clynes *et al*, 2000; Gennari *et al*, 2004; Arnould *et al*, 2006). Consistent with this view, Gennari *et al* (2004) demonstrated a correlation between tumour response to trastuzumab and *in vitro* ADCC in erbB2^+^ breast cancer patients receiving trastuzumab preoperatively. Similarly, Arnould *et al* (2006) showed that breast cancer patients who responded well to neoadjuvant trastuzumab and docetaxel had an increased tumour infiltration with lymphocytes, NK cells, and cytotoxic proteins such as granzyme B than patients who had a poor response.

The degree of ADCC, however, is variable even in HER2/neu overexpressors, and is mainly dependent on the number and function of NK cells (Varchetta *et al*, 2007). Natural killer cells are considered to be the best-suited lymphocytes for ADCC because they carry Fc*γ*RIII receptors, which are activator molecules, and not Fc*γ*RIIb receptors, which inhibit ADCC (Carson *et al*, 2001). Natural killer cells also carry receptors for IL-2, a cytokine that induces expansion of the NK cell population and enhances their function (Repka *et al*, 2003). Outpatient low-dose IL-2 has, therefore, been previously used to enhance cancer patients’ immune response to mAbs with little toxicity (Carson *et al*, 2001; Repka *et al*, 2003). Consistent with this, a significant increase in ADCC against USPC was detected after exposure of PBLs from healthy donors to low doses of IL-2 *in vitro* for a brief time (i.e., 5 h). These data, therefore, suggest that the administration of low, non-toxic doses of IL-2 *in vivo* may enhance the function of effector cells and increase the efficacy of pertuzumab therapy in USPC patients. Given the resistance of this tumour to standard anti-cancer therapy, these combined therapies might be particularly important in the treatment of USPC patients.

Both pertuzumab and trastuzumab induced significant ADCC against cell lines that express low HER2/neu levels, when compared with PBLs alone. However, the level of ADCC induced against these tumours was significantly lower than that detectable against USPC showing amplification of the *erbB2* gene by FISH. Surprisingly, in our experiments, when USPC cell lines that do not overexpress HER2/neu were subjected to a combination of pertuzumab and trastuzumab in a standard 5 h ^51^Cr release assay, the drug combination induced significantly higher cytotoxicity levels compared with control and either antibody used alone. The reason for this is not readily apparent, as both mAbs have a high affinity to the erbB2 receptor (Adams *et al*, 2006). Notably, in previous studies, pertuzumab was far more effective than trastuzumab in inhibiting the proliferation of breast cancer cell lines expressing varying levels of HER2/neu (Agus *et al*, 2002; Nahta *et al*, 2004). This was attributed to pertuzumab's ability to inhibit ligand-dependent heterodimerisation and activation of downstream targets such as mitogen-activated protein kinase and Akt. In addition, pertuzumab was found to inhibit ligand-dependent activation and signalling in several other cell lines (Jackson *et al*, 2004; Takai *et al*, 2005). Although a synergistic effect on signal transduction inhibition in those cell lines is certainly conceivable, it is not entirely clear how both antibodies would have a potentiating effect on ADCC, particularly in cell lines that do not overexpress HER2/neu. We are tempted to speculate that because pertuzumab and trastuzumab are known to recognise two different HER2/neu epitopes, the combined use of both antibodies may have caused a net increase in antibody binding to the surface of HER2/neu-positive tumour cells and, consequently, an increased tumour susceptibility to trastuzumab and pertuzumab-mediated ADCC. Flow cytometry data showing a significant increase in HER2/neu receptor expression on the surface of primary USPC cell lines using the combination of pertuzumab and trastuzumab when compared with both mAbs used alone are consistent with this hypothesis.

Our results also show that pertuzumab instigates CDC against USPC cells in culture. When human plasma (up to 50%) was added to the cytotoxicity assay, it produced an increase in the level of tumour cell lysis, most likely an effect of the complement proteins present in the plasma. Human plasma also contains endogenous IgG, and perhaps more importantly, the latter did not competitively inhibit or reduce the binding of pertuzumab with the low affinity Fc receptors on PBLs.

Previous reports investigating the anti-tumour effects of mAbs have shown that trastuzumab retains about 40% of its anti-tumour activity in Fc*γ*RIII−/− mice compared with wild-type mice, indicating that some biological effects of mAbs can be independent of Fc receptor binding (Clynes *et al*, 2000). Consistent with these data, in this study, we tested the potential cytostatic effect of pertuzumab to that of trastuzumab in multiple USPC primary cell lines expressing different levels of HER2/neu expression. Although both mAbs induced a significant inhibition in the proliferation of the primary USPC cell lines tested, regardless of their high or low HER-2/neu expression, pertuzumab was consistently found to be more effective than trastuzumab in inhibiting tumour proliferation. Finally, the combination of pertuzumab and trastuzumab was more cytostatic than trastuzumab mAb used alone. Taken together, these results further support a potential synergistic effect of the mAb combination on signal transduction inhibition in USPC.

In the clinic, trastuzumab (Herceptin) has been FDA approved as first-line therapy for metastatic breast cancer that overexpress HER2/neu (Slamon *et al*, 2001; Vogel *et al*, 2002). Given the high expression of HER2/neu in USPC, Villella *et al* (2006) and Santin *et al* (2008) have recently reported on the clinical use of trastuzumab, either as a single agent or in combination with chemotherapy, in patients with recurrent and/or chemotherapy-resistant USPC, with encouraging results. Indeed, in these clinical studies, a complete and a partial response were reported by Villella *et al* (2006) in two heavily pretreated USPC patients with the use of trastuzumab as a single agent, whereas Santin *et al* (2008) demonstrated a dramatic clinical improvement in two further USPC patients after the initiation of therapy with trastuzumab, with clinical responses confirmed by serial computerised tomography scan images and CA125 evaluations. Because pertuzumab was found to have similar pharmacokinetic and safety profiles to trastuzumab in animal models, it subsequently entered clinical development (Adams *et al*, 2006). In phase-I studies, pertuzumab was well tolerated, with the most common adverse events being fatigue, nausea, and vomiting (Agus *et al*, 2005). The maximum tolerated dose was not reached with dose escalation to 15 mg kg^−1^, and pharmacokinetic studies indicated a terminal elimination half-life of 2–4 weeks (Adams *et al*, 2006). Pertuzumab was studied in a phase-II multicentre trial in advanced, refractory ovarian cancer. In a total of 123 patients, there were five patients with a partial response, eight with stable disease, and ten with a decreased CA-125. Overall progression-free survival (PFS) was 6.6 weeks. erbB2 was overexpressed in 28% of patients. In this cohort, PFS was 20.9 weeks (Gordon *et al*, 2006).

In conclusion, we have shown for the first time that pertuzumab alone or in combination with trastuzumab may induce a high level of ADCC and is endowed with significant cytostatic effect against biologically aggressive USPC cell lines expressing varying levels of HER2/neu. On the basis of these results, we suggest including pertuzumab in phase-II protocols for patients with advanced, recurrent, and/or refractory USPC that overexpresses HER2/neu. The efficacy and safety of combining pertuzumab and trastuzumab in USPC tumours that do not overexpress erbB2 also warrant further investigation.

## Figures and Tables

**Table 1:** Patient characteristics from which the six USPC cell lines were established

**Patient**	**Age (years)**	**Race**	**Year of diagnosis**	**FIGO^a^ stage**	**USPC histopathology**
USPC ARK-1	62	AA	1997	IVA	Pure
USPC ARK-2	63	AA	1998	IVB	Pure
USPC ARK-3	59	AA	2006	IVB	Mixed
USPC ARK-4	73	C	2004	IVB	Pure
USPC ARK-5	73	AA	2006	IIIC	Pure
USPC ARK-6	62	C	2005	IB	Mixed

Abbreviations: AA=African American; C=Caucasian; USPC=uterine serous papillary adenocarcinoma.

a FIGO, International Federation of Gynecology and Obstetrics.

**Table 2:** HER2/neu expression in primary USPC cell lines

	**IHC**		**FISH**		**RT–PCR**
**Sample**	**Tissue**	**Cell Block**	**Tissue**	**Cell Block**	**mRNA copy number**
*Control*					1
USPC ARK-1	3+	3+	3.1	2.5	373
USPC ARK-2	3+	3+	6.3	5.2	607
USPC ARK-3	2+	3+	N/A	4.7	677
USPC ARK-4	1+	0	1.7	1.6	7
USPC ARK-5	1+	0	1.5	1.4	13
USPC ARK-6	1+	1+	N/A	0.9	6

Abbreviations: FISH=fluorescent *in situ* hybridisation; IHC=immunohistochemistry; RT–PCR=real-time PCR; N/A=not available for testing; USPC=uterine serous papillary adenocarcinoma.

Tissue refers to formalin-fixed, paraffin-embedded tissue blocks of the original tumour sample. Cell Block refers to formalin-fixed, paraffin-embedded cell blocks obtained from primary cell lines in culture.

**Table 3:** HER2/neu expression in primary USPC cell lines by flow cytometry

	**Pertuzumab^a^**		**Trastuzumab^a^**		**Combination^a^**		
**Sample**	**(%)**	**MFI**	**(%)**	**MFI**	**(%)**	**MFI**	***P*-value^b^**
USPC ARK-1	100	407.5	100	387.4	100	602.7	0.03
USPC ARK-2	100	1366	100	1381	100	1742	0.03
USPC ARK-3	100	607.5	100	679.7	100	751.8	0.04
USPC ARK-4	100	13.2	100	14.8	100	20.6	0.02
USPC ARK-5	100	31.7	100	30.7	100	45.5	0.04
USPC ARK-6	100	30.3	100	31.0	100	50.1	0.03

Abbreviations: MFI=mean fluorescence intensity; USPC=uterine serous papillary adenocarcinoma.

a Dose of pertuzumab and trastuzumab was 2.5 *μ*g ml^−1^. Total dose of the combination was 2.5 *μ*g ml^−1^.

b Unpaired samples *t*-test. Results of a matched flow cytometry experiment are shown and are representative of a minimum of three experiments for each USPC primary cell line. From each primary tumour sample 5000–10 000 gated cells were measured and analysed using a FACScalibur, using cell Quest software, as described in the Methods section.

**Table 4:** Antibody-dependent cellular cytotoxicity results in six USPC cell lines

**Sample**	**Control (%)**	**Rituximab (%)**	**Trastuzumab (%)^a^**	**Pertuzumab (%)^a^**	**Combination (%)^b^**
USPC ARK-1	1.6	4.5	44.7	52.8	54.2
USPC ARK-2	0.7	0.5	63.8	62	58.1
USPC ARK-3	0.6	1.9	67.3	66.7	68.8
USPC ARK-4	0.2	0.4	14.1	2.2	26.1
USPC ARK-5	4.7	3.7	34.5	17.7	41.1
USPC ARK-6	4.6	4.5	56.2	25.3	65.7

Abbreviation: USPC=uterine serous papillary adenocarcinoma.

a Dose of pertuzumab and trastuzumab was 2.5 *μ*g ml^−1^.

b Pertuzumab 1.25 *μ*g ml^−1^ and trastuzumab 1.25 *μ*g ml^−1^. The combination of pertuzumab and trastuzumab was significantly more effective in inducing antibody-dependent cell-mediated cytotoxicity in all three USPC cell lines that express low levels of HER2/neu when compared with the use of pertuzumab or trastuzumab used alone (Kruskal–Wallis test and *χ*^2^ analysis; *P*=0.001).

Results from a representative cytotoxicity experiment for each cell lines are shown.
